# Editorial: Roles of microbes in esophageal disease

**DOI:** 10.3389/fcimb.2023.1339579

**Published:** 2023-11-29

**Authors:** Learn-Han Lee, Xuefeng Gao, Gang Sun

**Affiliations:** ^1^ Sunway Microbiome Centre, School of Medical and Life Sciences, Sunway University, Sunway City, Malaysia; ^2^ Research Center for Life Science and Healthcare, China Beacons of Excellence Research and Innovation Institute (CBI), University of Nottingham Ningbo China, Zhejiang, China; ^3^ Department of Hematology and Oncology, International Cancer Center, Shenzhen Key Laboratory of Precision Medicine for Hematological Malignancies, Shenzhen University General Hospital, Shenzhen University Clinical Medical Academy, Shenzhen University Health Science Center, Shenzhen, China; ^4^ Central Laboratory, Shenzhen University General Hospital, Shenzhen, China; ^5^ Microbiota Division, Department of Gastroenterology and Hepatology, The First Medical Center, Chinese People’s Liberation Army (PLA) General Hospital, Beijing, China

**Keywords:** microbes, esophageal disease, microbiome, gastroesophageal reflux disease (GERD), eosinophilic esophagitis (EoE), esophageal squamous cell carcinoma (ESCC)

The human body is home to a vast ecosystem of microorganisms, collectively known as the microbiome, which plays a pivotal role in maintaining our health (Lim et al., 2022b). In recent years, research has shed light on the significance of the microbiome in various aspects of health (Kong et al., 2022), including its involvement in esophageal disease (ED) (Chen et al., 2022; Muszyński et al., 2022). This Research Topic aims to investigate the esophageal microbiota’s role in various EDs, such as gastroesophageal reflux disease (GERD), eosinophilic esophagitis (EoE) and esophageal squamous cell carcinoma (ESCC), shedding light on potential diagnostic and therapeutic avenues.

Two studies investigated the relationship between microbiota and GERD. The first study, focusing on proton pump inhibitors (PPIs), Shi et al. revealed mycobiota dysbiosis in GERD patients, irrespective of PPI use. PPI treatment exacerbated fungal dysbiosis, particularly with increased *Candida* colonisation, warranting further investigation for a deeper understanding of PPIs effects on fungal dynamics in GERD. The second study, Ye et al. examined gut microbiota in pediatric GERD, identifying imbalances in bacterial phyla and specific metabolic pathways related to arachidonic acid, tyrosine, glutathione, and caffeine. The study suggests potential therapeutic interventions targeting specific bacteria associated with these pathways. Together, these studies provide scientific insights into microbiota dysregulation in GERD, influencing our understanding and potentially reshaping therapeutic approaches for GERD. Meanwhile, in the third study, Zhang et al. scrutinised the intratumoral microbiome in ESCC. The study underscores their potential as prognostic indicators and therapeutic targets by linking higher microbiome diversity and *Lactobacillus* abundance to forming an immunosuppressive microenvironment. The findings highlight the symbiotic relationship between the intratumoral microbiome and the immune microenvironment, paving the way for innovative ESCC prognosis and treatment approaches.

Zou et al. comprehensively reviewed studies on esophageal microflora in GERD, EoE, ESCC and Barrett’s esophagus, suggesting a bidirectional causal relationship between ED and shifts in esophageal microflora. This implies that pathogenic microflora can reshape the mucosal microenvironment, and alterations in the mucosal microenvironment, in turn, can facilitate changes in the microflora. Meanwhile, Zhang et al. focused on reviewing EoE-related esophageal microbiome research, highlighting key findings such as alterations in microbial composition, the potential role of the microbiota in EoE pathogenesis through interactions with the epithelial barrier and immune system, modulation of the microbiota by EoE treatments, and the emerging therapeutic potential of targeting the esophageal microbiota. Nevertheless, despite the methodological challenges and existing limitations in esophageal microflora research, both Zou et al. and Zhang et al. indicate that the development of genomics and multi-omics approaches holds promise for enhancing our understanding of the esophageal microbiome, identifying molecular biomarkers for more accurate diagnosis, and ultimately contributing to the prevention and treatment of ED.

In conclusion, these studies underscore the oesophagal microbiota’s pivotal role in ED. From unraveling the complexities of EoE to questioning the implications of PPI use and exploring the intricate relationships in ESCC and pediatric GERD, the esophageal microbiota emerges as a dynamic player in the spectrum of ED. As we navigate this fascinating landscape, the call for larger, standardised studies becomes increasingly imperative to investigate the impact of interventions, such as targeted microbiota modulation (Lim et al., 2022a; Lim et al., 2022b; Sim et al., 2023), which holds promise for developing more effective therapeutic strategies in ED.

## Author contributions

L-HL: Conceptualization, Writing – original draft, Writing – review & editing. XG: Conceptualization, Writing – review & editing. GS: Conceptualization, Writing – review & editing.
